# Host–Pathogen Interaction 5.0

**DOI:** 10.3390/ijms252413596

**Published:** 2024-12-19

**Authors:** Andreas Burkovski

**Affiliations:** Microbiology Division, Friedrich-Alexander-Universität Erlangen Nürnberg, 91058 Erlangen, Germany; andreas.burkovski@fau.de; Tel.: +49-9131-85-28086

Microorganisms can interact with plants, animals, and humans in many different ways, e.g., beneficially as symbionts, indifferently as commensals, or harmfully as pathogens. The molecular basis of interaction between pathogens and their respective hosts is a focus of the Molecular Microbiology section of the *International Journal of Molecular Sciences*. To date, five Special Issues have been published. As in the preceding issues “Host–Pathogen Interaction”, “Host–Pathogen Interaction 2.0”, and “Host–Pathogen-Interaction 3.0” (summarized in [1]), “Host–Pathogen Interaction 4.0” and “Host–Pathogen-Interaction 5.0” aim to provide insights into the molecular interactions of bacteria, fungi, protozoa, and viruses with their respective hosts. 

Studies describing bacterium–host interactions accounted for the majority of submissions to the last two Special Issues, with an emphasis on the molecular mechanisms of Gram-negative bacteria. The delivery and role of E3 ubiquitin ligases from the NEL family, in respect to the reprogramming of host signaling, are reviewed by Bullones-Bolaños and coworkers [2]. The manipulation of the host by *Salmonella* is achieved by the Type III secretion-system-dependent effector protein SrfJ, a glucosylceramidase that alters the membrane composition and transcription patterns of host cells [3]. Potential host-specific determinants are analyzed in a comprehensive pangenome-wide analysis of *S. enterica* [4]. Among others, the identified genes encode proteins involved in secretion systems and surface structures. Molecular mechanisms controlling the host specificity of *Salmonella* interactions are also discussed in a review with applied focus, describing the use of *Salmonella*-based biorodenticides [5]. 

Other studies focusing on Gram-negative pathogens aim to characterize intercellular signaling pathways activated by *Helicobacter pylori* to stimulate inflammation and cancer development [6]; the interactions of the *Legionella pneumophila* lipopolysaccharide core region with host cell surface receptors to mediate adhesion and modulate immune responses [7]; the influence of *Leptospira* outer membrane proteins on adhesion [8]; and the influence of metal ions on the infection process of *Pseudomonas aeruginosa* in a zebrafish model [9].

Membrane vesicles in Gram-positive bacteria, their content and functions, and the influence of bacteria–host interactions are reviewed by Sangiorgio and coworkers [10], while the influence of bacterial communities on host responses to pharmaceuticals is discussed in [11]. These two topics, the delivery of bacterial molecules to eukaryotic host cells and the influence of microbiome composition on disease and treatment, are highly important new directions of research and may be a focus of future Special Issues.

As in the case of bacterial membrane vesicles, the extracellular vesicles (EVs) of eukaryotes have various functions. The EVs of the protozoan parasite *Naegleria fowleri* induce proinflammatory immune responses [12], an effect that has also been reported in Cathepsin Bs of this amoeba [13]. Other aspects of protozoan biology, pathogenesis, and epidemiology published in this Special Issue include a review of cellular and molecular mechanisms of *Plasmodium vivax* merozoite invasion into reticulocytes [14], the proteins forming the *Entamoeba histolytica* ESCRT-II complex during phagocytosis [15], and the development of *Babesia bovis* blood-stage vaccines [16].

In addition to bacteria and protozoa, fungi are important pathogens of plants and animals. Two articles submitted to “Host–Pathogen Interaction 4.0” and “Host–Pathogen Interaction 5.0” deal with post-penetration resistance against *Blumeria graminis forma specialis tritici*, the causative agent of powdery mildew in wheat [17], and Cla4A, a novel regulator of genomic expression in *Beauveria bassiana*, an insect-pathogenic fungus [18].

Lastly, viruses are important infectors of eukaryotes. Manuscripts were submitted that deal with the role of short linear motifs in the spike protein of SARS-CoV-2 in respect to immune system manipulation and evasion [19] and the role of lipid droplets during Zika virus infection in humans [20]. In addition, studies on bluetongue virus serotype 10 and 17 coinfection in ruminants [21], porcine epidemic diarrhea virus in pigs [22], and rabbit hemorrhagic disease viruses [23] are also included.

Taken together, this series of Special Issues receives continuing attention, reflecting the scientific interest and importance of this research field at the interface of molecular biology and medicine.
